# SOS Teeth: First Priority Teeth with Advanced Caries and Its Associations with Metabolic Syndrome among a National Representative Sample of Young and Middle-Aged Adults

**DOI:** 10.3390/jcm9103170

**Published:** 2020-09-30

**Authors:** Galit Almoznino, Ortal Kessler Baruch, Ron Kedem, Noam E. Protter, Boaz Shay, Nirit Yavnai, Dorit Zur, Eitan Mijiritsky, Itzhak Abramovitz

**Affiliations:** 1 Big Biomedical Data Research Laboratory, Hadassah School of Dental Medicine, Hebrew University, Jerusalem 91120, Israel; galit@almoznino.com; 2 Department of Endodontics, Hadassah School of Dental Medicine, Hebrew University, Jerusalem 91120, Israel; ortalbaruch1@gmail.com (O.K.B.); boaz@endo.co.il (B.S.); 3 Department of Oral Medicine, Sedation & Maxillofacial Imaging, Hadassah School of Dental Medicine, Hebrew University, Jerusalem 91120, Israel; 4 Medical Information Department, General Surgeon Headquarter, Medical Corps, Israel Defense Forces, Tel-Hashomer 02149, Israel; ron.kedem56@gmail.com (R.K.); Dorit48@mail.idf.il (D.Z.); 5 Forensic Unit, Medical Corps, Israel Defense Forces, Tel-Hashomer 02149, Israel; noamprotter@gmail.com; 6 Medical Research & Academy Section, Medical Corps, Israel Defense Forces, Tel-Hashomer 02149, Israel; nirityavnai@gmail.com; 7 Department of Otolaryngology, Head and Neck and Maxillofacial Surgery, Tel-Aviv Sourasky Medical Center, Sackler Faculty of Medicine, Tel Aviv 6139001, Israel; mijiritsky@bezeqint.net; 8 The Maurice and Gabriela Goldschleger, School of Dental Medicine, Tel-Aviv University, Tel Aviv 6139001, Israel

**Keywords:** caries, decayed teeth, SOS teeth, metabolic syndrome, hypertension, diabetes mellitus, hyperlipidemia, obesity, electronic medical record, electronic dental record

## Abstract

“SOS teeth” are defined as the first priority teeth for treatment, that have distinct cavitation reaching the pulp chamber or only root fragments are present. These are teeth with severe morbidity, that may require pulp capping, root canal treatment, or extraction, and therefore should be treated first. The study aims to explore whether or not a metabolic syndrome (MetS) is associated with SOS teeth. To that end, we performed across-sectional records-based study of a nationally representative sample of 132,529 military personnel aged 18–50 years, who attended the military dental clinics for one year. The mean number of SOS had no statistically significant association with: smoking (*p* = 0.858), alcohol consumption (*p* = 0.878), hypertension (*p* = 0.429), diabetes mellitus (*p* = 0.866), impaired glucose tolerance (*p* = 0.909), hyperlipidemia (*p* = 0.246), ischemic heart disease (*p* = 0.694), S/P myocardial infarction (*p* = 0.957), obstructive sleep apnea (*p* = 0.395), fatty liver (*p* = 0.074), S/P stroke (*p* = 0.589), and S/P transient ischemic attack (*p* = 0.095) and with parental history of: diabetes (*p* = 0.396)], cardiovascular disease (*p* = 0.360), stroke (*p* = 0.368), and sudden death (*p* = 0.063) as well as with any of the medical auxiliary examinations (*p* > 0.05). Cariogenic diet was positively associated with SOS teeth (*p* < 0.001). We conclude that SOS teeth had no statistically significant association with MetS components or with conditions that are consequences or associated with MetS. The only statistically significant parameter was a cariogenic diet, a well-known risk factor for caries and MetS.

## 1. Introduction

Dental caries is a diet-dependent, transmissible microbiologically mediated disease that follows an infectious and chronic disease model [1]. Dental caries is caused by the interrelationship of multiple factors over time which can be categorized into biological, behavioral, and environmental factors, which act upon the dentition throughout life [2]. To this day, dental caries is the most important oral disease and is of medical, social, and economic importance [3].

In our previous publication, we defined “SOS teeth” as the first priority teeth for treatment, that have distinct cavitation reaching the pulp chamber or only root fragments are present [4]. While the most commonly used epidemiological index for dental caries assessment [5] is the decayed missing filled surfaces/teeth (DMFS/T) index by the World Health Organization (WHO) [6], it has some limitations in dental caries assessment. While according to the DMF, a tooth with a dentin carious lesion is considered a diseased tooth [7], it fails to provide information regarding the clinical consequences of untreated dental caries, such as pulpal involvement, which may be more serious than the caries lesions themselves [8]. Moreover, assessment of SOS teeth, unlike the DMF, is done with X-ray imaging, which is important to identify “hidden caries” [9]. Given the global epidemic of untreated caries, assessment of first priority SOS teeth provides dentists and health authorities with useful information regarding urgent dental care needs to plan dental services [4]. In our previous publication, we assessed the prevalence and distribution by age and sex of SOS teeth among young to middle-aged adults [4]. In the current study, we intend to further explore the association between SOS teeth and metabolic syndrome (MetS) in this population. Another publication will analyze the associations of SOS with socio-demographic parameters, dental and orofacial morbidities.

Metabolic syndrome (MetS) is a cluster of metabolic risk factors for subsequent development of type 2 diabetes mellitus, cardiovascular diseases (CVD), and stroke [10]. The metabolic cluster is comprised of abdominal obesity, hypertension, hyperglycemia hypertriglyceridemia, and lowhigh-density lipoprotein (HDL) cholesterol levels [10]. There are several definitions for MetS [11,12], but the most broadly used is the National Cholesterol Education Program (NCEP) Adult Treatment Panel III (ATP III) criteria [10]. The prevalence of MetS was almost 25 percent in the adult US population, and the incidence of MetS increases with age [13,14,15]. MetS has been also linked with several obesity-related disorders such as fatty liver disease [16,17,18] and obstructive sleep apnea (OSA) [19].

A link between dental caries and cardiovascular diseases and MetS and its components was suggested in the literature, both in pediatric and adult populations [20,21,22]. Common risk factors of caries and MetS include, among others: age, race, low household income, weight, and abdominal obesity, smoking, alcohol consumption, carbohydrate-rich diet, and physical inactivity [23,24,25]. However, the association between advanced carious lesions, such as SOS teeth and MetS have not been studied yet to the best of our knowledge. In light of the English literature review, the primary objective of the study was to explore whether or not MetS components are associated with SOS teeth among young to middle-aged adults. To that end, we performed a cross-sectional records-based study of a nationally representative sample of 132,529 military personnel aged 18–50 years, who attended the military dental clinics for one year. We analyzed their socio-demographic, dental, and medical records. Specific objectives of the study were to study the associations and correlations of SOS teeth with: (1) Each of the MetS components and consequences and related conditions, including hypertension, diabetes mellitus, hyperlipidemia, impaired glucose tolerance (IGT), ischemic heart disease, status post (S/P) myocardial infarction, S/P stroke, and S/P transient ischemic attack (TIA), obstructive sleep apnea (OSA) and fatty liver; (2) metabolic parental medical history; (3) medical attendance patterns; (4) health-related risk factors; (5) auxiliary tests including blood tests used in the assessment of MetS components. We hypothesized that there is an association between SOS teeth and MetS. Exploring these associations is crucial to allow the appropriate implementation of strategies and literature-based interventions as well as to establish new research objectives that are essential for decision-makers policy to update procedures and current guidelines.

The military population in Israel is large, and constitutes a reliable source of data for epidemiologic surveys among young and middle-aged adults, partially because of the fact that conscriptionis mandatory in Israel for all Jewish, Druze, or Circassian citizens aged 18 and older [26]. It should be noted that medically complex patients serve in the IDF, in non-combat occupations, and even subjects deemed unfit for service for physical or mental health reasons can apply for volunteer positions [27]. Volunteers have the same access and rights to dental care as soldiers in mandatory service. Criteria for volunteering in the IDF for military exemption recipients are publicly available [28].

## 2. Methods

This is part of the Dental, Oral, Medical Epidemiological (DOME), which is a cross-sectional records-based big data study [4]. Socio-demographic, dental, and medical data were collected from a nationally representative sample composed of the Israel Defense Forces (IDF) military personnel who have undergone dental treatment at the dental clinics of the IDF between 1 January 2015, and 1 January 2016 [4]. We have previously described in detail the data collection from these databases [4].

### 2.1. Ethical Approval

The study is conforming to the STROBE guidelines and received approval from the Medical Corps Institutional Review Board (IRB), approval number: IDF-1281-2013. The IRB approved an exemption from written informed consent, owing to the retrospective study design that involves only records analysis.

### 2.2. Inclusion Criteria

Military personnel in mandatory or career service aged 18–50 years old with existing data regarding the subject in the CPR (a computerized patient record), DPR (Dental Patient Record), and the central demographic database.

### 2.3. Exclusion Criteria

Lack of socio-demographic, dental, or medical records regarding the subject in the CPR, DPR, and central demographic database records.

### 2.4. Study Variables

1. The following parameters were found in the central demographic database: sex and age.
2. The following parameters were found in the DPR: the number of SOS teeth.
3. The following parameters were found in the CPR: medical diagnoses and auxiliary tests including blood tests.

Data mining was done using the computerized databases and performed by the Medical Information Department, General Surgeon Headquarter, Medical Corps, Tel-Hashomer, Israel. The database is completely anonymous, as the data derived do not contain any identifying details.

### 2.5. The Dependent Variable: SOS Teeth

The teeth are the first priority for treatment, known as “teeth SOS”, and represent teeth that have a deep clinical and radiographic tooth decay that may require filling, root canal treatment, or extraction, as described in detail in our previous publication [4].

### 2.6. Independent Medical Parameters

#### 2.6.1. MetS Components and Consequences and Related Conditions

The parameters related to the general health status include the medical diagnoses of the patient as recorded in the CPR by the medical doctors (family physician/specialists). The following diagnoses were included: hypertension, diabetes mellitus, impaired glucose tolerance (IGT), hyperlipidemia, ischemic heart disease, status post (S/P) myocardial infarction, obstructive sleep apnea (OSA), fatty liver, S/P stroke, and S/P transient ischemic attack (TIA).

#### 2.6.2. Metabolic Parental Medical History

It was derived from the CPR and include the following self- reported parental medical history: diabetes mellitus, cardiovascular disease, stroke, and sudden death (yes/no question).

#### 2.6.3. Medical Attendance Patterns

The total number of appointments with a general physician- the number of attended medical appointments

#### 2.6.4. Assessment of Health-Related Habits

These include:

- Assessment of consumption of snacks and/or sweets between meals or instead of meals (yes/no) were found in the DPR as recorded by the dentist.
- Assessment of the current smoking status and alcohol consumption (yes/no) were found in the CPR as recorded by the general physician.

#### 2.6.5. Auxiliary Tests Including Blood Tests Used in the Assessment of MetS Components

They were drawn from the CPR as follows:

- Weight. Is measured as a routine by a medic before any medical examination.
- Systolic and diastolic blood pressure are measured as a routine by a medic before any medical examination, following five to ten minutes of rest in a sitting position, with an appropriately sized cuff on the right arm, at heart level using a manual sphygmomanometer, as detailed elsewhere [29].
- C-reactive protein (CRP). The prototypic acute-phase reactant C-reactive protein(CRP) has long been recognized as a useful marker and gauge of inflammation. CRP also plays an important role in host defense against invading pathogens as well as in inflammation [30]. CRP level of more than 3.0 mg/L increases the risk of cardiovascular disease (CVD) and a CRP level of lower than 1.0 mg/L decreases the risk of CVD [31].
- Glycated hemoglobin (HbA1c). Identifies the average plasma glucose concentration. Because red blood cells in the human body survive for 8–12 weeks before renewal, measuring glycated hemoglobin (or HbA1c) can be used to reflect average blood glucose levels over that duration, providing a useful longer-term gauge of blood glucose control. The normal value is below 5.7% [32].
- Glucose tolerance test:
  Blood glucose 0—Complete glucose value at 0 min during an oral glucose tolerance test (OGTT). Normal glucose tolerance after the immediate glucose load is lower than 110 mg/dL [33].
  Blood glucose 60—Complete glucose value at 60 min an OGTT. Glucose level below 180 mg/dL is considered normal [33].
  Blood glucose 120—Complete glucose value at 120 min an OGTT. Normal glucose tolerance two hours after a glucose load is lower than 140 mg/dL [33].
- Cholesterol. Complex mechanisms maintain cholesterol within physiological ranges and the dysregulation of these mechanisms results in embryonic or adult diseases, caused by either excessive or reduced tissue cholesterol levels [34]. Total cholesterol of 180 to 200 mg/dL or less is considered best; the acceptable borderline is 200–239 mg/dL [35].
- High-Density Lipoprotein (HDL)—HDL cholesterol efflux capacity that is impaired by inflammatory condition, serves as a predictor of prevalent and incident cardiovascular disease. [36]. Healthy individuals have levels above 40 mg/dL (1 mmol/L) in men and above 50 mg/dL (1.3 mmol/L) in women [37].
- Low-Density Lipoprotein (LDL)—Transports cholesterol from the liver to the tissues of the body. Elevated LDL levels are associated with an increased risk of cardiovascular disease. Healthy individuals have levels below 130 mg/dL (3.4 mmol/L) [38].
- LDL Cholesterol Calculated—Reflects the amount of cholesterol carried by LDL. The level of 100 mg/dL or below is considered ideal. Ranges of 100–129 mg/dL (2.6–3.3 mmol/L) is considered near ideal and 130–159 mg/dL is considered borderline high [39].
- Triglycerides—The major form of fat stored by the body that serves as the backbone of many types of lipids (fats). Triglycerides come from the food we eat as well as from being produced by the body. Normal levels are considered less than 150 mg/dL, the borderline high ranges from 150 to 199 mg/dL [40].
- Very low-density lipoprotein (VLDL)—Contains the highest number of triglycerides. Normal VLDL levels range from 5–40 mg/dL [41].
- Non-HDL Cholesterol—Multiple studies have shown that non-high-density lipoprotein cholesterol (non-HDL-C) is a better marker of cardiovascular risk than low-density lipoprotein cholesterol [42].

### 2.7. Statistical Analysis

Data were tabulated and statistical analyses were performed using SPSS software version 25.0 (IBM, Chicago, IL, USA). Statistical significance was considered as *P* < 0.05. Numerical variables are presented as means and standard deviations, categorical variables are presented as frequencies and percentages. Significance tests between SOS teeth and the independent variables included: non-paired *t*-test for categorical parameters and Pearson’s correlation for numerical variables.

## 3. Results

The study population included 132,529 patients who attended the dental clinics in 2015 with a mean age of 21.5 ± 5.5 and an age range of 18–50, including 99,466 (75.0%) males and 33,063 (25.0%) females. In our previous publication, we have shown that the prevalence of patients with SOS teeth was 9.16% (12,146/132,529) and the mean number of SOS teeth was 0.14 ± 0.52 [4]. We have also shown that the mean number of SOS teeth per patient had a statistically significant negative correlation with age and with male sex compared to females [4].

### 3.1. The Association of SOS Teeth with MetS Components, Consequences, and Related Conditions

Table 1 presents associations of SOS teeth with medical diagnoses related to MetS. There were no statistically significant associations between the mean number of SOS teeth and the following medical conditions: hypertension (*p* = 0.429), diabetes mellitus (*p* = 0.866), impaired glucose tolerance (*p* = 0.909), hyperlipidemia (*p* = 0.246), ischemic heart disease (*p* = 0.694), S/P myocardial infarction (*p* = 0.957), obstructive sleep apnea (*p* = 0.395), fatty liver (*p* = 0.074), S/Pstroke (*p* = 0.589), and S/P transient ischemic attack (*p* = 0.095) (Table 1).

### 3.2. The Association of SOS Teeth with Self-Reported Parental Medical History

Table 2 presents the association of the mean number of SOS teeth with self-reported parental medical history. There was no statistically significant association between the mean number of SOS teeth and any of the included parental medical histories: parental history of diabetes mellitus (*p* = 0.396), cardiovascular disease (*p* = 0.360), stroke (*p* = 0.368), and history of sudden death (*p* = 0.063) (Table 2).

### 3.3. The Association of SOS Teeth with Medical Attendance Patterns and Health-Related Habits

Table 3 presents the correlation of the mean number of SOS teeth with medical attendance patterns and health-related habits. A Pearson’s correlation was performed and demonstrated that SOS teeth had a statistically significant negligible negative correlation with the number of appointments with a general physician (Pearson’s correlation: −0.009; *p* = 0.002) (Table 3).

The mean number of SOS teeth had no statistically significant association with smoking (*p* = 0.858) and alcohol consumption (*p* = 0.878). The cariogenic diet was positively associated with the mean number of SOS teeth (*p* < 0.001) (Table 3).

### 3.4. The Correlations of SOS Teeth with Auxiliary Tests Including Blood Tests Used in the Assessment of MetS Components

Table 4 presents the correlations of SOS teeth with medical auxiliary examinations. A Pearson’s correlation test was performed and demonstrated that SOS teeth had no statistically significant correlation with any of the medical auxiliary examinations including the blood tests (*p* < 0.05) (see Table 4).

## 4. Discussion

SOS teeth are teeth that have deep clinical and radiographic tooth decay and are defined as the teeth to be treated first. To the best of our knowledge, this is the first study that assesses the association between metabolic syndrome and teeth with severe caries published in the English literature. Available studies that will be described below in the discussion, analyzed the associations between dental caries and MetS. The limitations of these studies is that they did not specifically analyze the associations of MetS with untreated advanced dental caries, which require urgent and more complicated treatment than restorative treatment. We included a large nationally representative sample of young to middle adults of the general population in Israel. In this study, we did not find associations between SOS teeth and the MetS components, consequences and related conditions as well as with the parental history related to metabolic morbidity, health-related habits, and relevant auxiliary tests. The only statically significant parameter was a cariogenic diet, a well-known risk factor for caries.

There is a limited body of research data available describing the SOS teeth. Since we could not find studies analyzing the association between advanced carious lesions and MetS to compare our results with other studies, we will assess our results with regards to available data on the association between dental caries in general and MetS.

### 4.1. The Association of General Health Status with the Mean Number of SOS Teeth

#### 4.1.1. Hypertension

In this study, there was no statistically significant association between the mean number of SOS teeth and hypertension, as well as with the measures of systolic and diastolic blood pressures. In line with our findings, a cross-sectional study demonstrated that the mean of DMFT score in normal, pre-hypertension, and hypertension groups were not statistically significant [43]. On the other hand, Johannssen et al. found that adolescents with a high prevalence of caries had higher blood pressure than caries-free adolescents [44]. An additional study found an association between caries and high blood pressure and stroke [45].

Another interesting explanation for the lack of association between hypertension and the mean number of SOS teeth could be attributed to reduced pain perception in spontaneous or experimentally induced highblood pressure, known as blood pressure-related hypoalgesia [46]. The finding was first described in Israel [47,48], and has since been described in many other studies [49,50,51,52,53,54,55]. A possible hypothesis is that higher pain thresholds could account for more non-attendance to dental appointments, and therefore these patients could not be captured in the study. However, the present study did not include assessment of pain thresholds and therefore we cannot test this hypothesis, and it should be tested in future studies that will include pain thresholds as co-variants.

#### 4.1.2. Diabetes Mellitus, HbA1C%, Fasting Glucose, and OGTT Values

Our study found that there was no statistically significant association between SOS teeth and diabetes mellitus as well as glycated hemoglobin (HbA1c), fasting glucose, and OGTT values. In agreement with our findings, a review by Taylor et al. concluded that the literature does not describe a consistent relationship between type 2 diabetes and dental caries [56]. However, others described an association between diabetes and an increase in caries in patients with a lack of metabolic control [57], such as type 2 diabetes mellitus patients hospitalized because of poor or worsened glycemic control [58].

#### 4.1.3. Hyperlipidemia and Serum Lipids Profile

In the present study, no significant association was found between the mean number of SOS teeth and hyperlipidemia. The literature shows conflicting results. A study on adolescents with cardiometabolic risk factors found significant correlations for decayed, missing, filled surfaces (DMFS) to levels of cholesterol, low-density lipoprotein, and triglycerides, compared to age-matched adolescents without cardio-metabolic risk factors [59]. On the other hand, a cross-sectional study of 13,998 participants aged 45–65 years found that caries was associated with metabolic syndrome and hyperglycemia, but not with abdominal obesity, hypertriglyceridemia, and elevated blood pressure [22].

#### 4.1.4. Cardiovascular Disease (CVD)

In the present study, no significant association was found between the mean number of SOS teeth and cardiovascular diseases. The results in the literature regarding the associations between the prevalence of caries and the presence of CVD are also conflicting. While no significant difference in the incidence of caries in patients with or without coronary heart disease was reported [60], there are studies that show an increased incidence of caries in children with congenital heart disease compared to age- and gender-matched healthy controls [61,62]. Significant associations were found between dental caries and CVD and its risk factors in the pediatric age group [63] and in elder subjects [44]. Although the association of dental caries with cardiovascular events remains controversial, a birth cohort confirmed the associations of dental caries with angina pectoris [21].

#### 4.1.5. Obstructive Sleep Apnea (OSA)

In the present study, no significant association was found between the mean number of SOS teeth and OSA. In line with our findings, a study conducted by Fouad et al. concluded that young children suffering from OSA and snoring had less dental caries, plaque deposition, and gingival inflammation and better oral hygiene [64]. Also, a study conducted by Acar et al. resulted in no correlation between OSA severity and the decayed, missing, and filled teeth index, leading to their conclusion that OSA does not negatively affect oral and dental health [65].

#### 4.1.6. Weight

In this study, it was found that there was no statistical correlation between the mean number of SOS teeth and weight. In the literature, the correlation between caries and weight, BMI, and obesity showed mixed results [66,67]. Willershausen et al. found among primary school pupils a significant correlation between a low BMI and the absence of carious lesions and a high BMI was linked to a high number of caries lesions [68]. Moreover, another study showed that adolescents with high BMI values had 1.6 times higher caries prevalence than those with lower BMI values [69]. Alswat et al. found a significant positive correlation between BMI and DMFT among adults in Saudi Arabia [70]. This positive association might be caused by an unhealthy lifestyle and poor carbohydrate-rich diet, which rise both dental caries and CVD risks [63]. On the other hand, Moreira et al. showed there was no statistically significant association between dental caries and obesity [71]. Also, Narksawat et al. [72] found that overweight children were less likely to have dental caries in primary and permanent dentition than normal-weight children. The underweight schoolchildren exhibited the highest sugar consumption that makes them at higher risk of developing dental caries, but they keep their weight by the high physical activity [73].

### 4.2. Health-Related Risk Habits and SOS Teeth

#### 4.2.1. Cariogenic Diet

This study found that the only statistically significant parameter associated with SOS teeth was a cariogenic diet, specifically the consumption of snacks and/or sweets between meals or instead of meals. Indeed, the assertion that diet plays a central role in the development of dental caries is unquestionable [74]. Observations in humans, in animals, and in vitro have shown clearly that frequent and prolonged oral exposure to certain carbohydrates is fundamental to caries activity. The effects of high sugar consumption were best revealed from the classic Vipeholm dental caries study which showed that sugar increased caries most if consumed between meals [75].

#### 4.2.2. Tobacco Smoking

Our findings demonstrated that smoking was not statistically associated with SOS teeth. The literature is replete with reports on tobacco smoking effects on oral health and on the other hand, other studies found no association between caries and smoking. Schmidt et al. reported that an increase in tobacco smoking was followed by a decrease in caries rate [76]. In contrast, Ludwick and Massler reported that those who smoked more than 15 cigarettes a day had a significantly higher number of decayed, missing, and filled teeth [77]. Smokers demonstrated a decreased buffering effect and possibly lower pH of the saliva and higher counts of Lactobacilli and Streptococcus mutans, which may indicate an increased susceptibility to caries [78]. In addition, various sugars and sweeteners are added intentionally during the tobacco manufacturing process of sugars [79]. On the other hand, the concentration of thiocyanate, a constituent of tobacco smoke, and normal saliva with possible caries-inhibiting effect were found to be higher in smoker’s saliva [78]. Therefore, one might predict fewer dental caries in smokers. The balance between these caries inhibiting versus caries promoting factors could account for the contradictory findings in the literature.

#### 4.2.3. Alcohol Consumption

In the present study, alcohol consumption showed no significant association with SOS teeth. This could be attributed to the low number of heavy consumers of alcohol (i.e., alcoholism) among military personnel. However, in a longitudinal study, excessive alcohol consumption was found to have a significant association with the number of caries lesions but not on periodontal conditions [80]. Also, individuals with the highest alcohol consumption visited dentists or dental hygienists more irregularly than individuals with a lower alcohol consumption which may have resulted in more decayed tooth surfaces, apical lesions, and calculus [80].

#### 4.2.4. Medical Attendance Patterns

In this study, there was a weak negligible correlation between SOS teeth and the total number of appointments with a general physician, which is in line with the findings that SOS teeth are not associated with more metabolic morbidities. To the best of our knowledge, the association between severe dental caries and general medical attendance patterns had not been published. However, dental attendance had been associated with caries. For example, a study conducted by Blinkhorn et al. showed that irregular attendees had significantly higher DMFT and fewer filled teeth. Both socioeconomic status and visiting behavior exerted significant independent effects on DMFT [81].

#### 4.2.5. Strengths and Limitations

The main strengths of the present study are the large sample size (12,146 subjects with SOS teeth and a total of 132,529 subjects comprising the study population) as well as the strict protocol utilizing dental and medical databases. Definitions were strict and uniform for all patients. For dental parameters, both clinical examination and radiographic assessment were included. Dental and medical attendance and medical indexes were derived from the records, and we did not rely on the patient’s report, which may be influenced by recall bias. Limitations of this study include the fact that there were some self-reported parameters, such as health-related behaviors and a history of parental morbidities, which could be influenced by recall bias. While the study evaluated contributing factors such as smoking, alcohol consumption, dietary habits, other health-related behaviors such as oral hygiene levels and physical activity levels were not analyzed. Because of the cross-sectional study design, we cannot assume causality, and therefore this paper only suggests associations and correlations between the variables. Additional studies, including long-term longitudinal population-based epidemiological surveys in other settings and populations, would help address these issues.

## 5. Conclusions

The goal of the present extensive nationally representative study was to analyze the association between SOS teeth and metabolic syndrome (MetS). SOS teeth had no statistically significant association with MetS components, consequences, and related conditions. The only statically significant parameter was a cariogenic diet, a well-known risk factor for caries, as well as for metabolic morbidity. Dentists and health authorities should be familiar with this data and integrate dental and general health promotion programs to focus on common risk habits such as dietary practices.

## Figures and Tables

**Table 1:** The association of the mean number of SOS teeth with metabolic syndrome components, consequences, and related conditions (non-paired *t* test).

		N	Mean Number of SOS Teeth	Std. Deviation	95% Confidence Interval for Mean		*p* Value
					Lower Bound	Upper Bound	
Hypertension	No	129,166	0.14	0.52	0.14	0.14	0.429
	Yes	3363	0.15	0.54	0.13	0.16	
	Total	132,529	0.14	0.52	0.14	0.14	
Diabetes Mellitus	No	132,184	0.14	0.52	0.14	0.14	0.866
	Yes	345	0.13	0.42	0.09	0.18	
	Total	132,529	0.14	0.52	0.14	0.14	
Impaired glucose tolerance (IGT) (IGT)	No	132,401	0.14	0.52	0.14	0.14	0.909
	Yes	128	0.13	0.54	0.04	0.23	
	Total	132,529	0.14	0.52	0.14	0.14	
Hyperlipidemia	No	131,568	0.14	0.52	0.14	0.14	0.246
	Yes	961	0.12	0.46	0.09	0.15	
	Total	132,529	0.14	0.52	0.14	0.14	
Ischemic heart disease	No	132,396	0.14	0.52	0.14	0.14	0.694
	Yes	133	0.12	0.42	0.05	0.19	
	Total	132,529	0.14	0.52	0.14	0.14	
S/P myocardial infarction	No	132,494	0.14	0.52	0.14	0.14	0.957
	Yes	35	0.14	0.55	−0.05	0.33	
	Total	132,529	0.14	0.52	0.14	0.14	
Obstructive sleep apnea (OSA)	No	132,211	0.14	0.52	0.14	0.14	0.395
	Yes	318	0.11	0.36	0.07	0.15	
	Total	132,529	0.14	0.52	0.14	0.14	
Fatty liver	No	131,591	0.14	0.52	0.14	0.14	0.074
	Yes	938	0.11	0.42	0.08	0.13	
	Total	132,529	0.14	0.52	0.14	0.14	
S/P stroke	No	132,437	0.14	0.52	0.14	0.14	0.589
	Yes	92	0.11	0.40	0.02	0.19	
	Total	132,529	0.14	0.52	0.14	0.14	
S/P transient ischemic attack (TIA)	No	132,430	0.14	0.52	0.14	0.14	0.095
	Yes	99	0.05	0.26	0.00	0.10	
	Total	132,529	0.14	0.52	0.14	0.14	

**Table 2:** The association of SOS teeth with self-reported parental medical history (* non-paired *t* test).

Parameter	Variable	N	Mean Number of SOS Teeth	Std. Deviation	95% Confidence Interval for Mean		*p* Value *
					Lower	Upper	
Diabetes in the family	No	132,163	0.14	0.52	0.14	0.14	0.396
	Yes	366	0.16	0.52	0.11	0.22	
	Total	132,529	0.14	0.52	0.14	0.14	
Cardiovascular disease in the family	No	131,907	0.14	0.52	0.14	0.14	0.360
	Yes	622	0.12	0.42	0.09	0.15	
	Total	132,529	0.14	0.52	0.14	0.14	
Family history of stroke	No	132,475	0.14	0.52	0.14	0.14	0.368
	Yes	54	0.07	0.26	0.00	0.15	
	Total	132,529	0.14	0.52	0.14	0.14	
Family history of sudden death	No	132,468	0.14	0.52	0.14	0.14	0.063
	Yes	61	0.26	0.85	0.04	0.48	
	Total	132,529	0.14	0.52	0.14	0.14	

**Table 3:** The correlations of SOS teeth with medical attendance patterns and health-related habits (* Pearson’s correlation, ^ non-paired *t* test).

Medical Attendance Patterns							
The number of appointments with a general physician		P value *			0.002		
		Pearson’s Correlation			−0.009		
**Health-related habits**		**N**	**Mean Number of SOS teeth**	**Std. Deviation**	**95% Confidence Interval for Mean**		***p*** **Value ^**
					**Lower Bound**	**Upper Bound**	
Smoking habits	No	125,645	0.14	0.52	0.14	0.14	0.858
	Yes	6884	0.14	0.48	0.13	0.15	
	Total	132,529	0.14	0.52	0.14	0.14	
Alcohol consumption	No	132,377	0.14	0.52	0.14	0.14	0.878
	Yes	152	0.13	0.58	0.04	0.23	
	Total	132,529	0.14	0.52	0.14	0.14	
Cariogenic diet	No	22,003	0.13	0.48	0.12	0.14	<0.001
	Yes	22,975	0.16	0.55	0.15	0.17	
	Total	44,978	0.14	0.52	0.14	0.15	

**Table 4:** The correlations of the mean number of SOS teeth with auxiliary tests including blood tests used in the assessment of MetS components (Pearson’s correlation).

Parameter	Mean ± SD	Pearson’s Correlation Test		
		N	Pearson’s Correlation	Sig. (2-Tailed)
Weight (kilograms)	73.29 ± 32.39	6414	−0.011	0.397
Systolic blood pressure (mmHg)	121.38 ± 13.82	11,244	0.018	0.060
Diastolic blood pressure (mmHg)	71.59 ± 13.12	11,244	−0.004	0.640
C reactive protein (CRP) (mg/L)	3.77 ± 10.19	2327	−0.004	0.859
Glycated hemoglobin (HbA1c) (%)	5.78 ± 1.14	164	−0.054	0.492
Fasting glucose (mg/dL)	87.12 ± 11.94	131	0.095	0.281
Glucose tolerance test- Glucose t0 (mg/dL)	89.68 ± 21.83	67	−0.103	0.406
Glucose tolerance test- Glucose t60	134.07 ± 46.36	96	−0.037	0.723
Glucose tolerance test- Glucose t120	107.32 ± 39.23	51	−0.105	0.464
Cholesterol (mg/dL)	175.83 ± 35.63	2062	0.002	0.910
High-density lipoprotein (HDL) (mg/dL)	48.28 ± 11.7	2062	−0.051	0.021
Low-density lipoprotein (LDL) (mg/dL)	108.34 ± 30.4	1400	0.010	0.721
LDL cholesterol calculated (mg/dL)	108.37 ± 30.41	1284	0.022	0.426
Triglycerides (mg/dL)	104.47 ± 64.04	2064	0.013	0.564
Very low-density lipoprotein (VLDL) (mg/dL)	20.61 ± 11.21	2059	0.004	0.872
Non-HDL cholesterol (mg/dL)	129.51 ± 35.01	1158	0.024	0.422

## Abbreviations

CPR	Clinical patient record
CRP	C-reactive protein
CVD	Cardiovascular disease
DMFT	decayed, missed, filled teeth
DPR	Dental patient record
HbA1c	Glycated hemoglobin
HDL	High-density lipoprotein
IGT	Impaired glucose tolerance
LDL	Low-density lipoprotein
MetS	Metabolic syndrome
OGTT	Oral glucose tolerance test
OSA	Obstructive sleep apnea
S/P	Status post
TIA	Transient ischemic attack
VLDL	Very low-density lipoprotein
